# Association of Bioelectrical Impedance Analysis Parameters with Malnutrition in Patients Undergoing Maintenance Hemodialysis: A Cross-Sectional Study

**DOI:** 10.3390/medicina61081396

**Published:** 2025-08-01

**Authors:** Minh D. Pham, Thang V. Dao, Anh T. X. Vu, Huong T. Q. Bui, Bon T. Nguyen, An T. T. Nguyen, Thuy T. T. Ta, Duc M. Cap, Toan D. Le, Phuc H. Phan, Ha N. Vu, Tuan D. Le, Toan Q. Pham, Thang V. Le, Thuc C. Luong, Thang B. Ta, Tuyen V. Duong

**Affiliations:** 1 Department of Nutrition, Military Hospital 103, Vietnam Military Medical University, Hanoi 12108, Vietnam; 2 International Ph.D. Program in Medicine, College of Medicine, Taipei Medical University, Taipei 11031, Taiwan; 3 Department of Infectious Diseases, Military Hospital 103, Vietnam Military Medical University, Hanoi 12108, Vietnam; 4 Faculty of Public Health, Haiphong University of Medicine and Pharmacy, Haiphong 04212, Vietnam; 5 Department of Nephrology and Hemodialysis, Military Hospital 103, Vietnam Military Medical University, Hanoi 12108, Vietnam; 6 Maxillofacial and Reconstructive Surgery Department, Military Hospital 103, Vietnam Military Medical University, Hanoi 12108, Vietnam; 7 Department of Microbiology, Faculty of Basic Medicine, Thai Nguyen University of Medicine and Pharmacy, Thai Nguyen 24117, Vietnam; 8 Department of Rheumatology and Endocrinology, Military Hospital 103, Vietnam Military Medical University, Hanoi 12108, Vietnam; 9 Director Office, Military Hospital 103, Vietnam Military Medical University, Hanoi 12108, Vietnam; 10 Cardiovascular Center, Military Hospital 103, Vietnam Military Medical University, Hanoi 12108, Vietnam; 11 Respiratory Center, Military Hospital 103, Vietnam Military Medical University, Hanoi 12108, Vietnam; 12 School of Nutrition and Health Sciences, Taipei Medical University, Taipei 11031, Taiwan

**Keywords:** percent body fat, skeletal muscle mass index, phase angle, ECW/TBW ratio, geriatric nutritional risk index, malnutrition, hemodialysis

## Abstract

*Background and Objectives:* Malnutrition is one of the most common complications in patients undergoing hemodialysis (HD) and is closely linked to increased morbidity and mortality. This study aimed to investigate the nutritional status of HD patients and the clinical relevance of bioelectrical impedance analysis (BIA) parameters such as the percent body fat (PBF), skeletal muscle mass index (SMI), extracellular water-to-total body water ratio (ECW/TBW), and phase angle (PhA) in assessing malnutrition in Vietnamese HD patients. *Materials and Methods:* This cross-sectional study was conducted among 184 patients undergoing hemodialysis in Hanoi, Vietnam. The BIA parameters were measured by the InBody S10 body composition analyzer, while malnutrition was assessed by the geriatric nutritional risk index (GNRI), with a GNRI <92 classified as a high risk of malnutrition. The independent BIA variables for predicting malnutrition and its cut-off values were explored using logistic regression models and a receiver operating characteristic (ROC) curve analysis, respectively. *Results:* Among the study population, 42.9% (79/184) of patients were identified as being at a high risk of malnutrition. The multivariate logistic regression analysis revealed that a higher ECW/TBW was independently associated with an increased risk of malnutrition, while the PBF, SMI, and PhA expressed significant and inverse associations with the malnutrition risk after adjusting for multiple confounders. The cut-off values for predicting the high risk of malnutrition in overall HD patients were determined to be 20.45%, 7.75 kg/m^2^, 5.45°, and 38.03% for the PBF, the SMI, the PhA, and the ECW/TBW ratio, respectively. *Conclusions:* BIA parameters, including the PBF, SMI, PhA, and ECW/TBW ratio, could serve as indicators of malnutrition in general Vietnamese patients with HD.

## 1. Introduction

The number of individuals receiving maintenance hemodialysis (HD) is steadily increasing worldwide [1]. In this population, malnutrition is a prevalent concern, with reported rates ranging from 7% to 90% depending on the specific population and nutritional assessment tools applied [2,3]. Malnutrition is defined as a condition caused by the insufficient intake or absorption of nutrients, resulting in changes in body compositions and a reduced cellular mass, which subsequently impair the physical capacity, mental function, and clinical outcomes [4]. In HD patients, malnutrition may arise from several contributing factors, including a poor dietary intake, metabolic imbalances, and the catabolic influence of dialysis therapy [5]. This condition is closely linked to increased healthcare costs, as well as higher rates of morbidity and mortality [6]. As such, the early recognition and accurate diagnosis of malnutrition are vital for improving clinical management and outcomes in the HD population.

In recent years, growing attention has been directed toward the clinical importance of nutritional management in patients with chronic kidney disease (CKD) [7]. A body composition assessment is a valuable supportive tool in evaluating the nutritional status, diagnosing malnutrition, and monitoring the effectiveness of nutritional interventions in CKD and across a wide range of other medical conditions [8]. Among the diverse methods available for the body composition evaluation that are well-known and frequently used for research, such as anthropometry, the tracer dilution method, densitometry, dual-energy X-ray absorptiometry (DEXA), air displacement plethysmography, magnetic resonance imaging (MRI), and computed tomography (CT), the bioelectrical impedance analysis (BIA) stands out as a preferred tool among nephrologists and clinical nutritionists. The BIA has been recognized for being rapid, reproducible, easy to perform; offering a safe and practical approach to quantifying body components; and containing the fat mass, muscle mass, and body water [7,9].

Several BIA-derived parameters have been reported to be significantly associated with the nutritional status of patients with HD [7,10,11,12]. Among these, the phase angle (PhA) has shown a significant inverse relationship with the risk of malnutrition. Patients with lower PhA values are more likely to develop protein–energy wasting (PEW); specifically, individuals with a PhA < 3.7° had a significantly higher risk of PEW compared to those with a PhA ≥ 5.0° [7]. In a prospective cohort study involving 375 HD patients across seven hospital dialysis centers, higher levels of body fat mass (FM) and percent body fat (PBF) were found to be associated with a reduced mortality risk [10]. The lean tissue index (LTI) value of HD patients was found to be positively associated with the serum albumin and protein intake [13,14]. Furthermore, advancements in BIA technology, such as the segmental multi-frequency bioelectrical impedance analysis (SMBIA), allow for more precise intracellular and extracellular water measurements. This technique generates an “edema index” calculated as the extracellular water-to-total body water (ECW/TBW) ratio, which indicates the fluid status. In a study of 158 HD patients, those with lower edema index values exhibited a better nutritional status based on the Subjective Global Assessment (SGA) [11]. A higher ECW/TBW ratio was associated with PEW and an increased risk of all-cause mortality [12]. These findings suggested that the ECW/TBW ratio may serve as a marker of nutritional deterioration and a prognostic indicator in HD patients.

In Vietnam, more than 8.7 million adults are affected by CKD, accounting for approximately 12.8% of the population. Around 30,000 end-stage renal disease (ESRD) patients have received dialysis treatment annually, meeting only 30% of the national demand for dialysis [15]. In this context, the assessment of BIA parameters in patients with HD could provide clinicians with accessible, non-invasive tools for the risk stratification of the nutritional status, which is valuable in routine outpatient care. The early identification of nutritional deficits through the BIA may facilitate timely interventions, potentially reducing complications and enhancing the management of malnutrition, thereby ultimately improving both the quality of life (QoL) and clinical outcomes [7,16,17]. Although the BIA has been investigated as a valuable method for detecting PEW in HD patients in various countries [16,17,18,19,20], its application in the Vietnamese HD population remains underexplored. Moreover, the generalizability of these findings remains uncertain due to ethnic differences in body composition [21]. Therefore, this study aimed to investigate the demographic characteristics, laboratory findings, BIA parameters, and nutritional status of patients undergoing HD. Additionally, we sought to examine the associations between several BIA-derived indicators, such as the SMI (skeletal muscle mass index), PBF, ECW/TBW ratio, and PhA, with malnutrition, while also identifying their optimal cut-off values for clinical prediction. We hypothesized that the SMI, PBF, ECW/TBW ratio, and PhA can predict the malnutrition status in Vietnamese HD patients.

## 2. Materials and Methods

### 2.1. Study Design and Population

**Figure 1:**
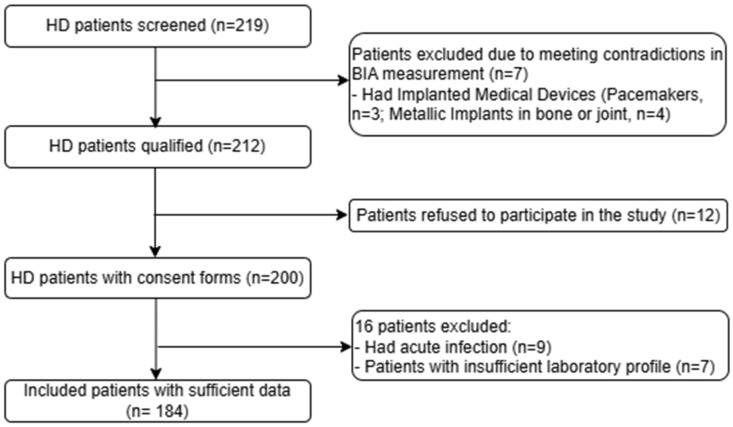
Study flow chart. HD: Hemodialysis.

This cross-sectional study was conducted between May 2023 and November 2023 at the Department of Nephrology and Hemodialysis of Military Hospital 103 in Hanoi, Vietnam. Adult patients aged over 18 years who had been undergoing HD for more than 3 months were recruited. A total of 219 HD patients were initially assessed for study inclusion. Patients were excluded when they had implanted medical devices, such as pacemakers or metallic implants, in bone or joint; had an acute infection; or had inadequate laboratory profiles. During recruitment, 12 patients did not consent to participate in the study. Finally, 184 patients with complete data were chosen for analysis (Figure 1). Informed written consent was obtained from all patients.

### 2.2. Measurements

#### 2.2.1. Demographic, Clinical, and Laboratory Parameters

All demographic and clinical data were collected from patients’ electronic medical records, including age, gender, the presence of comorbidities (hypertension, cardiovascular diseases, diabetes, and gout), and HD vintage. Age was categorized into 2 groups, younger and older adults, with a cut-off value of 60 years old, in accordance with previously published evidence [22]. HD vintage was identified as the duration from the patient’s first HD session to the study enrollment time. The patient’s blood samples were tested prior to the first hemodialysis session during the data collection week in accordance with standard hospital protocols. All laboratory testing was performed at the laboratory center of the hospital. The most recent laboratory parameters were retrieved from medical records for analysis. These included hematological results, liver enzymes (AST, aspartate aminotransferase; ALT, alanine aminotransferase), urea, creatinine, serum albumin, C-reactive protein (CRP), ferritin, and lipid profile components (cholesterol, triglyceride, LDL-C, low-density lipoprotein cholesterol; HDL-C, high-density lipoprotein cholesterol).

#### 2.2.2. Anthropometric and BIA Parameters

Participants’ body weight (in kg) and height (in cm) were measured at the time of admission by department staff. The body mass index (BMI) was derived as weight (kg) divided by height (m) squared (kg/m^2^). In this study, body composition analysis was conducted by a trained researcher using the portable InBody S10 analyzer (Biospace Co., Ltd., Seoul, Republic of Korea), a device previously utilized and validated in patients with CKD as well as those undergoing HD [17,23,24]. The measurements were collected on a dialysis day before the onset of the dialysis session, with the patient in a lying posture. Four conventional electrodes were attached to the patient in a supine position, two on the wrists and two on the ankles. All patients refrained from exercise within 24 h, avoided alcohol consumption within 48 h, and fasted immediately before the procedure to ensure measurement accuracy. The procedure and timing for BIA measurement were applied consistently for all participants and adhered strictly to the manufacturer’s instructions, with each measurement lasting approximately 3–5 min. Body composition parameters were collected, comprising soft lean mass (SLM), fat-free mass (FFM), skeletal muscle mass (SMM), percent body fat (PBF), body cell mass (BCM), visceral fat area (VFA), and skeletal muscle mass index (SMI). SMI was measured as the sum of the lean soft tissue of the two upper limbs and the two lower limbs divided by height squared (m^2^). Besides body composition metrics, body water compartments such as intracellular water (ICW), extracellular water (ECW), and total body water (TBW) were also assessed. The ECW/TBW ratio was expressed as a percentage, calculated by dividing ECW by TBW and multiplying by 100. Moreover, PA is an indicator of cellular health, membrane integrity, and hydration status, which was also recorded.

### 2.3. Nutritional Risk Assessment

In our study, nutritional risk was evaluated using the geriatric nutritional risk index (GNRI), a simple and widely utilized tool for assessing nutritional status in various clinical settings, such as both elderly populations [25] and HD patients [26,27]. The GNRI was calculated using the following formula:

GNRI = (1.489 × serum albumin (g/L) + (41.7 × (body weight/ideal body weight))

where ideal body weight (kg) was defined as (height in meters)^2^ × 22 (kg/m^2^). If a patient’s actual body weight exceeded their ideal body weight, the weight-to-ideal weight ratio was set to 1.0, following standard practice. A lower GNRI value indicates a higher risk of malnutrition. Based on previous literature, the study patients were categorized into the high risk of malnutrition with GNRI < 92 and the low risk of malnutrition with GNRI ≥ 92 [26,27,28]. 

### 2.4. Statistical Analysis

Continuous values were expressed as mean, standard deviation (SD) for normally distributed data or median, and interquartile range (IQR) for non-normally distributed data. Categorical variables were presented as numbers (percentages). Group comparisons between males and females and between high and low malnutrition risk groups were performed using independent samples *t*-tests for continuous variables and Chi-squared tests for categorical variables. When the assumption of normality was not met, the Mann–Whitney U test was used to assess differences in continuous variables. The correlations between laboratory and BIA parameters with GNRI were evaluated using the Spearman test.

Logistic regression analysis was carried out to identify factors associated with malnutrition risk, including demographic, clinical, laboratory, and body composition variables. As previous studies have suggested that the factors associated with nutritional status may differ by gender [29,30,31], we conducted analyses in the total population and stratified by sex (male and female). The multivariable logistic regression model included variables with a *p*-value < 0.05 in univariate analysis. Age, gender, comorbidity, and HD vintage were found in prior studies to be associated with malnutrition in HD patients [32,33,34], which can confound the association between laboratory, BIA parameters, and malnutrition risk. Therefore, these factors were adjusted in the multivariate models. Logistic analysis results were expressed as odds ratio (OR) with 95% confidence interval (CI).

In addition, a receiver operating characteristic (ROC) curve analysis was conducted to evaluate diagnostic performance and determine gender-specific cut-off values for BIA indicators. The optimal thresholds were determined based on the maximum Youden index and the smallest distance to (0, 1), which combines sensitivity and specificity [35]. The Youden index, calculated as sensitivity (1-specificity), ranges from 0 to 1, with higher values reflecting superior diagnostic accuracy. While the distance metric was computed as [(1 − sensitivity)^2^ + (1 − specificity)^2^], with the point on the ROC curve closest to (0, 1) representing better diagnostic performance [35]. A *p*-value < 0.05 was considered statistically significant. All statistical analyses were tested using SPSS 22.0 (SPSS software, SPSS Inc., Armonk, NY, USA).

## 3. Results

### 3.1. Clinical Characteristics of the Study Population

Of the 184 patients, 89 (48.4%) were older than 60 years old, and 35.3% of subjects had at least one comorbidity. The means of the height, weight, and BMI were 159.8, 55.1, and 21.5, respectively. The data for patients with a high risk of malnutrition accounted for 42.9% (79/184). The median HD vintage was 36 months (Table 1).

Regarding hematological parameters, no significant differences were observed between the low and high malnutrition risk groups in the neutrophil percentage, lymphocyte percentage, RBC count, or platelet count. However, the high-risk group demonstrated significantly lower levels of the WBC count, hemoglobin, and hematocrit compared to the low-risk group. Additionally, serum creatinine and urea concentrations were also significantly decreased in the high-risk group (*p* < 0.05). Patients categorized as high-risk for malnutrition showed significantly lower levels of serum albumin and triglycerides and higher HDL-C levels compared with their counterparts. There were no significant differences between the two groups in terms of liver enzyme levels (AST, ALT), CRP, total cholesterol, LDL-C, and ferritin (Table 1).

With regard to body composition parameters, patients classified as high-risk of malnutrition exhibited a significantly lower muscle mass (SLM, FFM, SMM, SMI). They also showed a significantly reduced PBF, VFA, and BCM. In addition, these patients had a lower body water content (ICW, ECW, TBW) along with a significantly decreased PhA. Conversely, patients in the high-risk group had higher ECW/TBW ratios than those at low nutritional risk (*p* < 0.05) (Table 1).

Clinical characteristics of the study population according to gender are presented in Supplementary Table S1.

### 3.2. Associations Between the Biomarkers and Malnutrition Risk of the Study Patients

The results of the univariate logistic regression analysis evaluating the associations between clinical variables and the risk of malnutrition in the overall study population, as well as in male and female subgroups, are presented in Supplementary Table S2. Continuous independent variables with *p*-values < 0.05 in the univariate logistic regression model were tested with a correlation matrix prior to their inclusion in the multivariate analysis (Supplementary Table S3). Table 2 illustrates the findings of the multivariate logistic regression examining the associations between laboratory and BIA parameters with the malnutrition risk of the overall study patients, men, and women.

In particular, the univariate regression results presented significant negative correlations between the levels of hemoglobin, hematocrit, triglyceride, and the malnutrition risk in overall patients and in males (*p* < 0.05) but not in females (Supplementary Table S2). In the multivariate logistic regression, these associations remained significant for hemoglobin and hematocrit in the overall participants and males, while triglyceride levels showed a consistent inverse relationship with the malnutrition risk across all groups, including females (Table 2).

### 3.3. Associations Between BIA Parameters and the Malnutrition Risk of the Study Patients

#### 3.3.1. Associations Between Muscle-Fat Parameters and Malnutrition Risk

Body composition indices containing the SLM, FFM, SMM, PBF, BCM, VFA, and SMI were observed to be negatively correlated with the risk of malnutrition across the overall subjects, as well as in both men and women, according to the univariate logistic regression analysis (*p* < 0.05) (Supplementary Table S2). These variables were subsequently analyzed using multivariate logistic regression models.

Focusing on the lean mass and muscle-related indicators, the adjusted logistic regression results revealed that higher values of SLM, FFM, SMM, and SMI were significantly linked to a lower risk of malnutrition, particularly among females. Specifically, the SMM and SMI were strong predictors in the overall sample (aOR = 0.870, 95%CI = 0.813–0.931, *p* < 0.001; aOR = 0.647, 95%CI = 0.500–0.837, *p* = 0.001, respectively), with the SMI showing a notably strong association in females (aOR = 0.436, 95%CI = 0.238–0.799, *p* = 0.007) but not reaching significance in males. Regarding fat mass and cellular integrity parameters, increased levels of PBF, BCM, and VFA values were also inversely associated with the malnutrition risk in the total subjects and both sexes (*p* < 0.05) (Table 2).

#### 3.3.2. Association Between ECW/TBW Ratio and Malnutrition Risk

The univariate logistic regression findings displayed a significant positive correlation between a higher ECW/TBW ratio and an increased malnutrition risk in the total subjects, males, and females (*p* < 0.05) (Supplementary Table S2). In the multivariate models, after adjusting for confounding factors such as the age, comorbidity, and HD vintage, this association remained statistically significant in the total population (aOR = 2.162, 95%CI = 1.575–2.968, *p* < 0.001), in men (aOR = 3.510, 95%CI = 1.928–6.388, *p* < 0.001), and in women (aOR = 1.630, 95%CI = 1.120–2.371, *p* = 0.011) (Table 2). 

#### 3.3.3. Association Between PhA and Malnutrition Risk

In the unadjusted analysis, the PhA exhibited a significant negative association with the risk of malnutrition across the total sample, as well as in both male and female subgroups (*p* < 0.05) (Supplementary Table S2). This inverse relationship persisted in adjusted models, where the PhA was a significant indicator of malnutrition in the overall population (aOR = 0.481, 95%CI = 0.338–0.686, *p* < 0.001), in males (aOR = 0.385, 95%CI = 0.218–0.680, *p* = 0.001), and females (aOR = 0.612, 95%CI = 0.382–0.980, *p* = 0.041), even after the full adjustment for clinical variables (Table 2).

### 3.4. Cut-Off Values of BIA Parameters to Detect High Risk of Malnutrition in HD Patients

**Figure 2:**
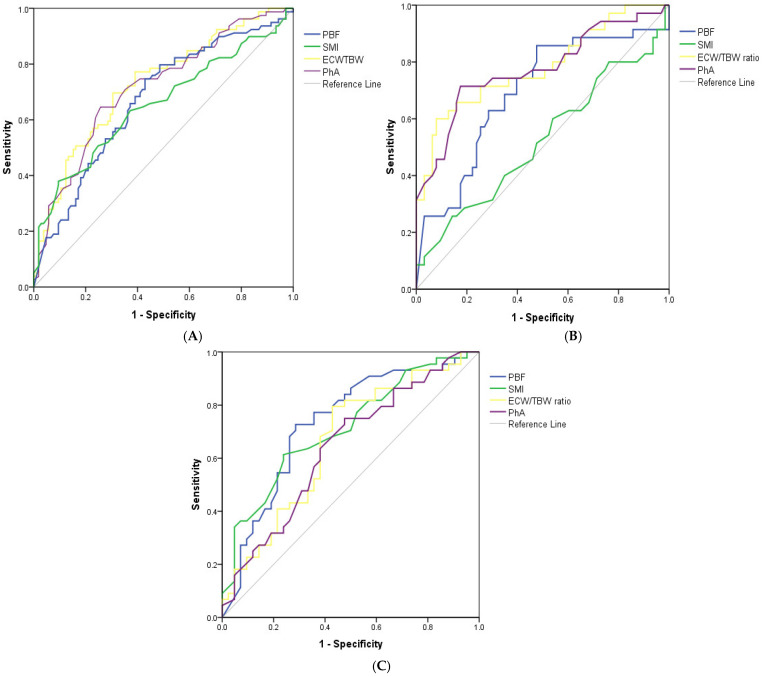
ROC curve analysis for BIA parameters to detect malnutrition in overall HD patients (**A**), males (**B**), and females (**C**). Abbreviations: ROC, receiver operating characteristic; PBF, percent body fat; SMI, skeletal muscle mass index; ECW/TBW, extracellular water-to-total body water; and PhA, phase angle.

Based on the correlation matrix results (Supplementary Table S3), four key BIA-derived parameters were identified as the most representative independent indicators of the malnutrition risk in HD patients, comprising the PBF, SMI, ECW/TBW ratio, and PhA. Their respective cut-off values and diagnostic performance metrics are shown in Table 3. Overall, these indicators demonstrated acceptable performance in predicting the high risk of malnutrition. In particular, the ECW/TBW ratio and PhA showed superior diagnostic performances compared to the other parameters in both the overall sample and the male subgroup (AUC = 0.728 and 0.717, respectively, in the overall population; AUC = 0.782 and 0.775, respectively, in males). Conversely, in the female subgroup, the PBF and SMI showed greater diagnostic accuracy, with an AUC = 0.733 for the former and an AUC = 0.712 for the latter (Figure 2 and Table 3).

The optimal cut-off points, determined using the Youden index, were consistent with those obtained by minimizing the distance to the (0, 1) point on the ROC curve. Another noteworthy point is that the SMI performed poorly as a predictor of the malnutrition risk in HD males, with an AUC = 0.523 (Figure 2 and Table 3).

## 4. Discussion

To the best of our knowledge, this is the first study in Vietnam to assess the nutritional status of HD patients using the GNRI and to examine the associations between BIA parameters and the malnutrition risk in this population. The findings highlighted a positive association between the ECW/TBW ratio and the risk of malnutrition, while the SMI, PBF, and PhA were found to be negatively associated with the likelihood of malnutrition. Additionally, this study revealed gender-specific differences in BIA-related factors linked to the malnutrition risk, as well as distinct threshold values for these indicators in predicting the nutritional risk.

A variety of tools have been developed to assess malnutrition in patients undergoing HD, comprising the Malnutrition Inflammation Score (MIS), SGA, BMI, and criteria from the International Society of Renal Nutrition and Metabolism (ISRNM), as well as the GNRI [6]. In this study, the GNRI was utilized to evaluate the nutritional status of HD patients, which is an objective and composite nutritional screening tool that incorporates serum albumin and the ratio of the actual to ideal body weight. This tool was initially developed to predict the morbidity and mortality risk in hospitalized older adults [25], which has since been validated and widely used in patients with CKD, especially those undergoing HD [26,27]. Among the available objective nutritional assessment methods, the GNRI is considered one of the simplest and most reliable indices for identifying the malnutrition risk [36]. Previous studies conducted in Vietnam have primarily relied on the SGA or BMI to assess the nutritional status in HD populations [34]. Compared with the SGA or BMI, the GNRI offers a more standardized, objective, and reproducible assessment based only on laboratory and anthropometric data that are routinely collected in clinical settings [37]. Given its practicality and accuracy, we recommend implementing the GNRI in dialysis centers across Vietnam or other low- and middle-income countries (LMICs) as a routine tool for evaluating the nutritional status of HD patients.

Interestingly, we found that RBC counts did not differ significantly between high- and low-risk malnutrition groups, while values of hemoglobin and hematocrit were significantly lower in the high-risk group. This discrepancy may be explained by the reduced plasma volume and lower ECW in the high-risk group (12.0 L for the high-risk group vs. 13.2 L for the low-risk group; *p* = 0.005), which can artificially elevate hemoglobin and hematocrit levels. These findings align with a prior study showing a negative correlation between ECW and hemoglobin in CKD patients [38]. In this study, we observed a significant relationship between hemoglobin, an anemia-related parameter, and the malnutrition risk in HD patients. Specifically, those classified as high-risk for malnutrition demonstrated significantly lower levels of hemoglobin compared to the low-risk group (90.3 and 83.6 g/L, respectively). Reduced hemoglobin may arise from iron deficiency, chronic inflammation, or inadequate erythropoiesis, all of which are exacerbated by malnutrition [39]. Our findings are consistent with the prior research [32], while other studies have reported no statistically significant association between hemoglobin levels and malnutrition [33,40,41]. Such differences may be attributable to variations in the sample size, population characteristics, and the sensitivity or specificity of the malnutrition classification tools employed across studies. Moreover, in our analysis, the level of hemoglobin was witnessed to be negatively associated with the risk of malnutrition in the overall HD sample in the adjusted analysis (aOR = 0.980, *p* = 0.022). Anemia is a prevalent complication in the HD population and is regarded as a major contributor to morbidity. Its pathophysiological is linked to a diminished appetite, and heightened catabolic activity may further promote the development of PEW [42,43]. These findings underscore the complex and bidirectional relationship between anemia and malnutrition, reinforcing the need for a comprehensive, multidisciplinary approach to patient management.

In addition to the biochemical parameters, this study provided valuable insights into the body composition characteristics of adult HD patients, as assessed by the BIA. Our study showed a significant decrease in ECW among HD patients with a high risk of malnutrition, which aligns with some previous studies [12,44]. While ECW levels have been reported to be elevated in malnourished HD patients [45]. The observed decrease in ECW in our cohort may reflect the loss of lean body mass, as ECW is predominantly distributed in skeletal muscle. Consequently, muscle wasting in malnourished individuals leads to a proportional decline in both ICW and ECW. Additionally, the chronic inflammation associated with malnutrition may impair endothelial function, thereby reducing effective ECW retention in the interstitial space [46]. Further investigations involving larger cohorts are needed to validate and expand upon these findings.

Among other BIA parameters, we found that patients at a high risk of malnutrition exhibited a significantly lower SMI, PBF, PhA, and a higher ECW/TBW ratio compared to those at low risk of malnutrition (*p* < 0.001). The results were in line with the prior studies. A study in Brazil reported that sarcopenia and malnutrition patients had a significantly reduced SMI, body fat, and PhA [47], while another study found that HD patients with a better nutritional status based on the SGA had lower ECW/TBW ratios [11]. Furthermore, our multivariate regression findings showed that there was a significant negative correlation between the SMI and risk of malnutrition (aOR = 0.647, *p* = 0.001 for overall patients and aOR = 0.436, *p* = 0.007 for females). Decreases in the SMI have previously been identified as reliable indicators of sarcopenia in patients with pre-dialysis CKD, HD, and kidney transplant recipients (KTRs) [7]. A prior study also suggested that malnutrition contributes to the development of sarcopenia in HD patients by decreasing the muscle mass, strength, and physical performance [48]. Therefore, the early management of malnutrition may be critical in preventing sarcopenia in this population.

Notably, we did not find a significant association between the SMI and malnutrition risk in males in adjusted models (aOR = 0.845, *p* = 0.445). We hypothesize that the SMI may serve as a more sensitive indicator of the malnutrition risk in females than in males. This may be attributed to the naturally higher muscle mass and SMI in males due to hormonal and physiological factors and typically greater physical activity levels [31,49,50]. While females have a lower baseline muscle mass, changes are more clinically meaningful. Thus, variations in the SMI among males may not reflect the malnutrition status as distinctly as in females. However, this observation warrants further investigation and validation in future studies.

Regarding the ECW/TBW, this ratio has been widely recognized as an indicator of the volume status in HD patients and has been shown to be positively associated with malnutrition [11,12,51]. In a study by Takahiro Yajima et al., a higher ECW/TBW ratio was significantly correlated with increased protein–energy wasting (PEW) scores among patients with HD [12]. Consistent with these findings, our study also demonstrated a positive association between the ECW/TBW ratio and malnutrition risk in the total study population (aOR = 2.162, *p* < 0.001), in females (aOR = 1.630, *p* = 0.011), and more strongly among male patients (aOR = 3.501, *p* < 0.001). The potential pathophysiological mechanism underlying this association may involve fluid overload, inducing bowel wall edema, which facilitates the translocation of endotoxins from the gut into the systemic circulation. This process can trigger systemic inflammation, contributing to protein catabolism and hypoalbuminemia, which are hallmarks of malnutrition. In turn, hypoalbuminemia may further exacerbate fluid retention and systemic edema, creating a vicious cycle of inflammation and nutritional deterioration [12].

Another key BIA parameter that has been reported in a number of previous studies is the PhA, which reflects the integrity of cell membranes and the distribution of intracellular and extracellular water, with higher values typically indicating a better cell function and nutritional status [52]. This parameter has been proposed as a valuable prognostic nutritional marker in various populations, including patients with CKD not yet on dialysis, those undergoing HD [16,18,53], as well as in pediatric [54] and oncology settings [55]. Particularly, a study in Malaysian HD patients identified the PhA as an independent predictor of PEW (aOR = 0.308, *p* = 0.001) [18]. Similarly, in patients with advanced CKD awaiting kidney transplantation, Muñoz-Redondo et al. found that a PhA ≤ 4.85° demonstrated a significantly positive association with malnutrition, even after adjusting for patient characteristics (aOR = 3.8; 95% CI: 1.1–13.8; *p* = 0.042) [24]. In line with previous studies, our results illustrated a significant inverse association between the PhA and the risk of malnutrition in the general population (aOR = 0.481, *p* < 0.001), as well as in males (aOR = 0.385, *p* < 0.001) and in females (aOR = 0.612, *p* < 0.001). The results suggest that individuals with higher PhA values were less likely to be malnourished. This relationship may be explained by the fact that malnutrition is a pathological state marked by the loss of fat and muscle reserves, which subsequently impairs the normal function of healthy cells by disrupting their membrane integrity and physiological function [18]. Since the PhA captures early alterations in cellular health, it may serve as a sensitive marker for early malnutrition and could be useful in evaluating the effectiveness of nutritional interventions, even before changes are detectable by traditional methods such as the SGA [56].

Additionally, our analysis identified a PhA cut-off value of 5.45° for detecting a high malnutrition risk in the overall HD population. Establishing reference values for the PhA and other key BIA-derived indicators for the prediction of malnutrition in Vietnamese HD patients is crucial for their effective and safe application in routine clinical nutritional assessments. Notably, the PhA threshold observed in this study is higher than those reported in earlier research, where cut-offs ranged from 3.7° to 4.7° [16,18,20]. This discrepancy may stem from differences in patient demographics (e.g., age, gender, ethnicity) and variations in BIA devices and measurement frequencies (single vs. multi-frequency) [17,18]. In addition to those factors, we believe that variations in the methods used to classify malnutrition may also contribute to differences in the cut-off values of the predictive indicators. While this study employed the GNRI, prior studies predominantly used the ISRNM diagnostic framework for PEW or malnutrition classification [16,17,18]. The timing of the BIA measurement should also be considered a potential factor influencing variations in the PhA and body water parameters among HD patients. Previous research has demonstrated that ECW, ICW, and TBW values decrease significantly following dialysis compared to pre-dialysis levels [57].

In this research, the PhA cut-off value of 5.45° expressed a good diagnostic performance, with a sensitivity of 70.9%, a specificity of 64.8%, and an AUC of 0.717. This sensitivity is slightly lower than that reported in other studies (77.7–86.4%) [17,19], while it is higher than the 62.5% reported in a Malaysian cohort [18]. This variation may reflect differences in methods used to determine the optimal cut-off value. Unlike previous studies, we applied both the Youden index and the closest distance to (0,1) method to refine the threshold estimation, enhancing the robustness of our results.

Our study has some limitations. First, this study was conducted at a single center with a relatively small sample size, which may limit the generalizability of findings. Additionally, the cross-sectional design restricts the ability to establish causal relationships between associated factors and malnutrition. It is challenging to discern whether changes in the body composition, such as an elevated ECW/TBW ratio or a reduced PhA, lead to a decline in the GNRI or whether malnutrition is the driving factor behind these alterations. Second, several important clinical variables—such as underlying diseases, medication use, hemodialysis treatment characteristics, and dietary intake data, which are essential components of a comprehensive nutritional assessment in HD individuals—were not included in the analysis. Their omission may have influenced both the study outcomes and the interpretation of the findings. Third, the GNRI is primarily designed to estimate the risk of malnutrition rather than to serve as a definitive diagnostic tool like the PEW criteria proposed by the ISRNM. Moreover, this assessment relies on the serum albumin and weight, which can be skewed by the fluid status. In particular, albumin levels are known to be influenced by inflammation, infection, and hepatic function, in addition to the nutritional status. We attempted to minimize the impact of inflammatory states by excluding patients with acute infections. However, it is important to note that chronic inflammation is prevalent in HD patients and may reduce albumin levels independently of the nutritional status [58]. Fourth, BIA parameters were collected at a single time point during enrollment, and longitudinal changes over time were not assessed, which limits insights into the progression or improvement of the nutritional status. Despite these limitations, our study has several strengths, such as being the first study in Vietnam describing BIA characteristics and investigating BIA-derived factors associated with malnutrition in patients with HD. In addition, the nutritional status was assessed using the GNRI, a validated and objective index that is particularly suitable for use in resource-limited settings. Furthermore, the associated BIA parameters of malnutrition and their cut-off values were explored not only in the total population but were also stratified by sex and adjusted for multiple confounding factors known to influence the nutritional risk. These methodological considerations enhance the significance and clinical relevance of our findings.

## 5. Conclusions

Our study demonstrated that the SMI, PBF, PhA, and ECW/TBW could be used as markers to reflect the nutritional status in patients with maintenance HD. Incorporating these body composition variables into routine nutritional assessments may enhance the early detection, management, and prognostication of malnutrition in this population. Future research should include prospective multicenter studies with larger cohorts and participants from varied ethnic backgrounds to enhance the validity and generalizability of our findings.

## Figures and Tables

**Table 1:** Patient characteristics according to GNRI group (*n* = 184).

Parameters	Total (*n* = 184)	GNRI		*p*-Value
		GNRI ≥ 92 (*n* = 105)	GNRI < 92 (*n* = 79)	
Age				0.025
<60 years	95 (51.6)	62 (59.0)	33 (41.8)	
≥60 years	89 (48.4)	43 (41.0)	46 (58.2)	
Gender				0.035
Male	119 (64.7)	63 (60.0)	35 (44.3)	
Female	65 (35.3)	42 (40.0)	44 (55.7)	
Comorbidity				0.514
No	119 (64.7)	70 (66.7)	49 (62.0)	
One or more	65 (35.3)	35 (33.3)	30 (38.0)	
Height (cm)	159.8 (7.2)	160.5 (7.4)	159.0 (7.6)	0.192
Weight (kg)	55.1 (9.4)	58.8 (8.7)	50.2 (7.9)	<0.001
BMI (kg/m^2^)	21.5 (2.8)	22.8 (2.5)	19.8 (2.2)	<0.001
HD vintage (months)	36 (12–60)	36 (12–72)	24 (4–60)	0.023
Laboratory findings				
WBC (×10^3^/µL)	6.1 (5.0–7.7)	6.7 (5.5–7.9)	5.3 (4.5–7.2)	0.001
Neutrophils (%)	65.2 (10.2)	65.5 (11.0)	64.7 (9.0)	0.603
Lymphocytes (%)	20.6 (7.9)	20.3 (8.3)	20.8 (7.2)	0.676
RBC (×10^6^/µL)	2.85 (0.63)	2.93 (0.64)	2.75 (0.61)	0.160
Hemoglobin (g/L)	87.4 (19.1)	90.3 (20.6)	83.6 (16.3)	0.019
Hematocrit (%)	26.2 (5.7)	27.1 (6.1)	25.2 (5.1)	0.025
Platelet (×10^3^/µL)	195.5 (152.0–233.0)	199.0 (157.0–231.0)	187.0 (142.0–236.0)	0.547
AST (IU/L)	17.6 (13.8–26.4)	16.6 (13.0–23.7)	18.8 (15.8–28.5)	0.055
ALT (IU/L)	13.1 (8.8–20.0)	11.9 (8.9–19.2)	13.9 (8.9–20.9)	0.578
Serum albumin (g/L)	39.0 (36.9–41.7)	40.2 (38.8–43.0)	37.1 (34.8–38.8)	<0.001
Urea (mmol/L)	23.7 (17.0–28.4)	24.7 (18.8–30.0)	20.1 (16.6–27.2)	0.013
Creatinine (µmol/L)	854.5 (698.1–1049.3)	919.0 (790.4–1114.7)	750.2 (662.3–936.5)	<0.001
CRP (mg/L)	3.9 (1.5–10.9)	3.4 (1.6–13.5)	5.5 (1.6–9.5)	0.933
Cholesterol (mmol/L)	4.57 (1.09)	4.64 (1.14)	4.48 (1.01)	0.360
Triglyceride (mmol/L)	1.70 (1.08–2.56)	2.04 (1.31–2.80)	1.29 (0.89–1.96)	<0.001
LDL-C (mmol/L)	2.88 (0.79)	2.96 (0.92)	2.77 (0.75)	0.128
HDL-C (mmol/L)	0.98 (0.81–1.18)	0.92 (0.79–1.05)	1.10 (0.90–1.23)	0.002
Ferritin (ng/mL)	182.5 (78.5–434.2)	123.9 (77.1–443.4)	210.6 (113.9–406.6)	0.399
BIA parameters				
SLM (kg)	42.4 (37.3–49.3)	44.5 (39.1–50.8)	38.8 (34.5–47.0)	<0.001
FFM (kg)	45.0 (40.0–52.3)	47.1 (41.7–53.8)	41.4 (37.4–49.6)	<0.001
SMM (kg)	24.6 (21.3–29.2)	25.9 (22.7–29.7)	22.1 (19.5–27.1)	<0.001
PBF (%)	16.9 (10.3–24.3)	20.5 (14.0–25.5)	14.5 (7.4–18.1)	<0.001
BCM (kg)	29.2 (25.6–34.3)	30.7 (27.1–34.8)	26.5 (23.6–32.0)	<0.001
VFA (cm^2^)	39.9 (23.5–57.2)	44.1 (26.1–65.7)	33.6 (19.0–46.7)	0.003
SMI (kg/m^2^)	7.2 (6.4–8.0)	7.4 (6.9–8.1)	6.8 (5.9–7.7)	<0.001
ICW (L)	20.4 (17.9–23.9)	21.4 (18.9–24.3)	18.5 (16.5–22.3)	<0.001
ECW (L)	12.8 (11.3–14.6)	13.2 (11.6–14.9)	12.0 (10.8–14.0)	0.005
TBW (L)	33.2 (29.1–38.3)	34.7 (30.5–39.6)	30.4 (27.5–36.7)	<0.001
ECW/TBW (Total,%)	38.6 (37.3–39.6)	39.2 (38.4–40.2)	38.2 (37.1–38.9)	<0.001
PhA (°)	5.40 (4.63–6.40)	4.80 (4.15–5.70)	5.90 (5.10–6.70)	<0.001

Categorical variables were presented as *n* (%). Normally distributed continuous data were demonstrated as mean and standard deviation (SD). Skewed continuous data were presented as median and interquartile range (IQR). Abbreviations: GNRI, geriatric nutritional risk index; BMI, body mass index; HD, hemodialysis; WBC, white blood cell; RBC, red blood cell; AST, aspartate aminotransferase; ALT, alanine aminotransferase; CRP, C-reactive protein; LDL-C, low-density lipoprotein cholesterol; HDL-C, high-density lipoprotein cholesterol; BIA, bioelectrical impedance analysis; SLM, soft lean mass; FFM, fat free mass; SMM, skeletal muscle mass; PBF, percent body fat; BCM, body cell mass; VFA, visceral fat area; SMI, skeletal muscle mass index; ICW, intracellular water; ECW, extracellular water; TBW, total body water; ECW/TBW, extracellular water-to-total body water ratio; and PhA, phase angle.

**Table 2:** The multiple logistic regression analysis for the determination of the association between laboratory, BIA parameters and a high risk of malnutrition in the overall sample (*n* = 184), males (*n* = 98), and females (*n* = 86).

Parameters	High Risk of Malnutrition					
	Overall Sample		Males		Females	
	aOR (95% CI)	*p*-Value	aOR (95% CI)	*p*-Value	aOR (95% CI)	*P*-Value
Laboratory parameters						
Hemoglobin, 1 g/L increase	0.980 (0.964–0.997)	0.022	0.974 (0.951–0.997)	0.025	0.993 (0.969–1.019)	0.613
Hematocrit, 1% increase	0.941 (0.890–0.994)	0.031	0.925 (0.858–0.996)	0.039	0.978 (0.896–1.067)	0.620
Urea, 1 mmol/L increase	0.958 (0.922–0.995)	0.026	0.928 (0.873–0.988)	0.019	0.977 (0.930–1.026)	0.347
Creatinine, 1 µmol/L increase	0.998 (0.996–0.999)	0.001	0.997 (0.995–0.999)	0.008	0.999 (0.997–1.001)	0.195
Triglyceride, 1 mmol/L increase	0.619 (0.463–0.828)	0.001	0.628 (0.433–0.912)	0.014	0.595 (0.363–0.977)	0.040
HDL-C, 1 mmol/L increase	11.731 (2.897–47.506)	0.001	16.099 (7.139–81.124)	<0.001	3.055 (0.519–17.983)	0.217
BIA parameters						
SLM, 1 kg increase	0.923 (0.884–0.962)	<0.001	0.931 (0.865–1.001)	0.055	0.851 (0.764–0.948)	0.003
FFM, 1 kg increase	0.926 (0.890–0.964)	<0.001	0.934 (0.872–1.001)	0.051	0.857 (0.774–0.950)	0.003
SMM, 1 kg increase	0.870 (0.813–0.931)	<0.001	0.868 (0.769–0.980)	0.023	0.764 (0.640–0.911)	0.003
PBF, 1 percent increase	0.921 (0.885–0.959)	<0.001	0.914 (0.861–0.970)	0.003	0.885 (0.824–0.950)	0.001
BCM, 1 kg increase	0.880 (0.828–0.936)	<0.001	0.878 (0.786–0.981)	0.022	0.781 (0.665–0.918)	0.003
VFA, 1 cm^2^ increase	0.974 (0.960–0.989)	0.001	0.975 (0.956–0.995)	0.014	0.961 (0.934–0.989)	0.012
SMI, 1 kg/m^2^ increase	0.647 (0.500–0.837)	0.001	0.845 (0.549–1.302)	0.445	0.436 (0.238–0.799)	0.007
ICW, 1 L increase	0.833 (0.763–0.910)	<0.001	0.830 (0.708–0.973)	0.021	0.703 (0.558–0.885)	0.003
ECW, 1 L increase	0.823 (0.718–0.942)	0.005	0.907 (0.727–1.131)	0.386	0.660 (0.472–0.921)	0.015
TBW, 1 L increase	0.905 (0.857–0.955)	<0.001	0.919 (0.837–1.008)	0.072	0.817 (0.712–0.937)	0.004
ECW/TBW, 1% increase	2.162 (1.575–2.968)	<0.001	3.510 (1.928–6.388)	<0.001	1.630 (1.120–2.371)	0.011
PhA, 1 degree increase	0.481 (0.338–0.686)	<0.001	0.385 (0.218–0.680)	0.001	0.612 (0.382–0.980)	0.041

Results obtained after adjusting for age, comorbidity, and HD vintage. Abbreviations: aOR, adjusted odds ratio; 95% CI, 95% confidence interval; HDL-C, high-density lipoprotein cholesterol; BIA, bioelectrical impedance analysis; SLM, soft lean mass; FFM, fat-free mass; SMM, skeletal muscle mass; PBF, percent body fat; BCM, body cell mass; VFA, visceral fat area; SMI, skeletal muscle mass index; ICW, intracellular water; ECW, extracellular water; TBW, total body water; ECW/TBW, extracellular water-to-total body water ratio; and PhA, phase angle.

**Table 3:** Cut-off values of BIA parameters to detect high risk of malnutrition in HD patients.

	Cut-Off Value *	Cut-Off Value ^#^	AUC	95%CI	Sensitivity (%)	Specificity (%)	*p*-Value
Overall sample (*n* = 184)							
PBF, %	20.45	20.45	0.672	0.593–0.751	79.7	50.5	<0.001
SMI, kg/m^2^	7.75	7.75	0.654	0.572–0.736	77.2	36.2	<0.001
ECW/TBW, %	38.63	38.63	0.728	0.654–0.801	69.6	69.5	<0.001
PhA, °	5.45	5.45	0.717	0.642–0.792	70.9	64.8	<0.001
Males (*n* = 98)							
PBF, %	17.45	17.45	0.691	0.577–0.805	85.7	47.6	0.002
SMI, kg/m^2^	8.25	8.25	0.523	0.397–0.648	65.7	31.7	0.711
ECW/TBW, %	39.31	39.31	0.782	0.681–0.883	60	92.1	<0.001
PhA, °	5.15	5.15	0.775	0.670–0.879	71.4	82.5	<0.001
Females (*n* = 86)							
PBF, %	21.1	21.1	0.733	0.624–0.841	72.7	64.3	<0.001
SMI, kg/m^2^	6.7	6.7	0.712	0.604–0.820	77.3	47.6	0.001
ECW/TBW, %	38.39	38.39	0.660	0.543–0.776	79.5	57.1	0.011
PhA, °	5.65	5.65	0.640	0.523–0.757	75.0	52.4	0.026

* The optimal cut-off points determined by the maximum Youden index value. ^#^ The optimal cut-off points determined by the point closest to (0, 1). Abbreviations: AUC, area under the curve; 95%CI, 95% confidence interval; PBF, percent body fat; SMI, skeletal muscle mass index; ECW/TBW, extracellular water-to-total body water ratio; and PhA, phase angle.

## Data Availability

The data supporting the findings of this study can be obtained from the corresponding author upon reasonable request.

## Supplementary Materials

The following supporting information can be downloaded at https://www.mdpi.com/article/10.3390/medicina61081396/s1: Table S1: Patient characteristics according to gender (n = 184), Table S2: Univariate logistic regression analysis for the determination of the association between clinical parameters and a high risk of malnutrition in the total subjects (*n* = 184), males (*n* = 98), and females (*n* = 86), and Table S3: Correlations between independent continuous variables with *p* < 0.05 in the univariate logistic regression and GNRI in overall subjects (*n* = 184).
