# Supplementary material for: Association of Bioelectrical Impedance Analysis Parameters with Malnutrition in Patients Undergoing Maintenance Hemodialysis: A Cross-Sectional Study
Source: Medicina (Kaunas). 2025 Aug 1;61(8):1396. doi: 10.3390/medicina61081396 (PMC12388193; doi:10.3390/medicina61081396)
Supplement: Supplementary file 1 [file medicina-61-01396-s001.zip › medicina-3721747-supplementary.pdf]

Supplementary data for the following study:

Association of bioelectrical impedance analysis parameters with malnutrition in patients undergoing maintenance hemodialysis: A cross-sectional study

Supplementary Table S1: Patient characteristics according to gender (*n*=184)

Parameter	Total (*n*=184)	Male (*n*=98)	Female (*n*=86)	*p*-value
Age				0.375
< 60 years	95 (51.6)	54 (55.1)	41 (47.7)	
≥ 60 years	89 (48.4)	44 (44.9)	45 (52.3)	
Comorbidity				0.282
No	119 (64.7)	67 (68.4)	52 (60.5)	
One or more	65 (35.3)	31 (31.6)	34 (39.5)	
Height(cm)	159.8 (7.2)	164.8 (5.6)	154.2 (5.0)	<0.001
Weight (kg)	55.1 (9.4)	60.4 (8.0)	49.1 (6.8)	<0.001
BMI (kg/m^2)	21.5 (2.8)	22.3 (2.7)	20.6 (2.7)	<0.001
HD vintage (months)	36 (12-60)	24 (12-60)	36 (6-72)	0.723
Laboratory findings				
WBC (×10^3/μL)	6.1 (5.0-7.7)	6.1 (4.9-7.8)	6.0 (5.0-7.3)	0.760
Neutrophils (%)	65.2 (10.2)	64.6 (9.9)	65.8 (10.6)	0.445
Lymphocytes (%)	20.6 (7.9)	20.8 (8.1)	20.3 (7.6)	0.770
RBC (×10^6/μL)	2.85 (0.63)	2.84 (0.64)	2.85 (0.63)	0.990
Hemoglobin(g/L)	87.4 (19.1)	89.3 (20.2)	85.3 (17.6)	0.159
Hematocrit (%)	26.2 (5.7)	26.8 (6.2)	25.6 (5.1)	0.178
Platelet (×10^3/μL)	195.5 (152.0-233.0)	187.0 (140.0-232.0)	200.5 (157.0-234.0)	0.273
AST (IU/L)	17.6 (13.8-26.4)	16.4 (13.4-27.9)	18.1 (15.8-24.5)	0.521
ALT (IU/L)	13.1 (8.8-20.0)	12.8 (8.5-22.7)	13.2 (9.2-18.4)	0.973
Serum albumin (g/L)	39.0 (36.9-41.7)	39.4 (36.9-42.4)	38.6 (36.6-40.6)	0.132
Urea (mmol/L)	23.7 (17.0-28.4)	22.9 (17.8-27.4)	24.6 (16.8-29.9)	0.854
Creatinine (μmol/L)	854.5 (698.1-1049.3)	919.5 (754.7-1090.5)	772.2 (633.7-943.2)	<0.001
CRP (mg/L)	3.9 (1.5-10.9)	3.5 (1.5-9.1)	4.9 (1.7-13.0)	0.516
Cholesterol (mmol/L)	4.57 (1.09)	4.38 (1.13)	4.80 (0.99)	0.012
Triglyceride (mmol/L)	1.70 (1.08-2.56)	1.67 (0.99-2.76)	1.78 (1.15-2.23)	0.920
LDL-C (mmol/L)	2.88 (0.79)	2.75 (0.82)	3.04 (0.73)	0.019
HDL-C (mmol/L)	0.98 (0.81-1.18)	0.95 (0.77-1.11)	1.02 (0.87-1.23)	0.013
Feritin (ng/mL)	182.5 (78.5-434.2)	218.2 (103.8-321.7)	146.6 (79.9-427.4)	0.510
BIA parameters				
SLM, kg	42.4 (37.3-49.3)	48.0 (44.4-52.1)	37.5 (33.8-39.2)	<0.001
FFM, kg	45.0 (40.0-52.3)	50.9 (47.0-55.0)	39.9 (35.9-41.9)	<0.001
SMM, kg	24.6 (21.3-29.2)	27.9 (25.7-30.5)	21.4 (19.2-22.9)	<0.001
PBF, %	16.9 (10.3-24.3)	15.0 (7.8-22.0)	19.2 (14.1-25.4)	0.004
BCM, kg	29.2 (25.6-34.3)	32.9 (30.5-35.7)	25.7 (23.3-27.3)	<0.001
VFA, cm^2	39.9 (23.5-57.2)	34.2 (16.4-60.1)	42.2 (30.2-57.1)	0.125
SMI, kg/m^2	7.2 (6.4-8.0)	7.8 (7.4-8.4)	6.4 (5.8-7.0)	<0.001

ICW, L	20.4 (17.9-23.9)	23.0 (21.3-24.9)	17.9 (16.3-19.1)	<0.001
ECW, L	12.8 (11.3-14.6)	14.2 (13.0-15.6)	11.3 (10.1-12.0)	<0.001
TBW, L	33.2 (29.1-38.3)	37.4 (34.7-40.6)	29.3 (26.3-30.5)	<0.001
ECW/TBW (Total,%)	38.57 (37.30-39.59)	38.45 (37.18-39.40)	38.68 (37.79-39.80)	0.057
PhA (°)	5.40 (4.63-6.40)	5.65 (4.80-6.70)	5.30 (4.40-6.10)	0.016
GNRI	99 (94-103)	101 (96-106)	98 (93-101)	0.010

Categorical variables were presented as *n* (%). Normally distributed continuous data were demonstrated as mean and standard deviation (SD). Skewed continuous data were presented as median and interquartile range (IQR)

Abbreviations: BMI, body mass index; HD, hemodialysis; WBC, white blood cell; RBC, red blood cell; AST, aspartate aminotransferase; ALT, alanine aminotransferase; CRP, C-reactive protein; LDL-C, low-density lipoprotein cholesterol; HDL-C, high-density lipoprotein cholesterol; SLM, soft lean mass; FFM, fat-free mass; SMM, skeletal muscle mass; PBF, percent body fat; BCM, body cell mass; VFA, visceral fat area; SMI, skeletal muscle mass index; ICW, intracellular water; ECW, extracellular water; TBW; ECW/TBW, extracellular water-to-total body water ratio; PhA, phase angle, GNRI, geriatric nutritional risk index.

Supplementary Table S2: Univariate logistic regression analysis for the determinant of the association between clinical parameters and a high risk of malnutrition in the total subjects (*n*=184), males (*n*=98), and females (*n*=86)

**Parameters**	**High risk of malnutrition**					
	**Total subjects**		**Males**		**Females**	
	**OR (95 %CI)**	***p*-value**	**OR (95 %CI)**	***p*-value**	**OR (95 %CI)**	***p*-value**
Age						
<60 years	Ref		Ref		Ref	
≥60 years	2.011 (1.111-3.635)	0.021	2.609 (1.116-6.096)	0.027	1.447 (0.619-3.386)	0.394
Gender						
Male	Ref					
Female	1.886 (1.044-3.406)	0.035	-	-	-	-
Comorbidity						
No	Ref		Ref		Ref	
Yes	1.224 (0.666-2.252)	0.515	1.477 (0.615-3.550)	0.383	1.080 (0.455-2.564)	0.862
HD vintage, 1 month increase	0.993 (0.985-1.001)	0.083	0.995 (0.983-1.006)	0.345	0.990 (0.979-1.002)	0.09
WBC, 1×10^3/μL increase	0.940 (0.837-1.054)	0.289	0.946 (0.805-1.113)	0.504	0.933 (0.790-1.103)	0.416
Neutrophils, 1% increase	0.992 (0.964-1.021)	0.601	1.001 (0.960-1.044)	0.953	0.980 (0.941-1.021)	0.343
Lymphocytes, 1% increase	1.008 (0.971-1.046)	0.674	0.984 (0.933-1.037)	0.537	1.042 (0.983-1.103)	0.165
RBC, 1×10^6/μL increase	0.629 (0.329-1.203)	0.161	0.523 (0.203-1.351)	0.181	0.724 (0.289-1.813)	0.491
Hemoglobin, 1g/L increase	0.981 (0.965-0.997)	0.021	0.975 (0.954-0.997)	0.027	0.991 (0.968-1.016)	0.487
Hematocrit, 1% increase	0.941 (0.892-0.993)	0.027	0.928 (0.864-0.997)	0.041	0.970 (0.892-1.055)	0.479
Platelet, 1×10^3/μL increase	0.998 (0.993-1.003)	0.367	0.995 (0.988-1.001)	0.112	1.003 (0.994-1.013)	0.498
AST, 1 IU/L increase	1.017 (0.989-1.047)	0.237	1.035 (0.988-1.085)	0.144	0.999 (0.958-1.041)	0.945
ALT, 1 IU/L increase	1.005 (0.985-1.026)	0.618	1.029 (0.990-1.069)	0.145	0.989 (0.959-1.020)	0.481
Urea, 1mmol/L increase	0.956 (0.922-0.992)	0.017	0.928 (0.874-0.985)	0.015	0.975 (0.930-1.023)	0.304
Creatinine, 1μmol/L increase	0.998 (0.996-0.999)	<0.001	0.997 (0.995-0.999)	0.003	0.998 (0.997-1.001)	0.114
CRP, 1mg/L increase	0.998 (0.983-1.014)	0.834	1.010 (0.982-1.039)	0.475	0.990 (0.966-1.014)	0.399
Cholesterol, 1mmol/L increase	0.875 (0.658-1.163)	0.358	0.876 (0.598-1.285)	0.499	0.751 (0.469-1.205)	0.235
Triglyceride, 1mmol/L increase	0.671 (0.509-0.886)	0.005	0.688 (0.485-0.975)	0.036	0.642 (0.399-1.034)	0.068
LDL-C, 1mmol/L increase	0.736 (0.496-1.093)	0.129	0.642 (0.369-1.118)	0.117	0.719 (0.381-1.355)	0.308
HDL-C, 1mmol/L increase	5.620 (1.638-19.285)	0.006	11.990(1.787-30.580)	0.011	2.351 (0.450-12.282)	0.311
Ferritin, 1ng/mL increase	1.001 (0.999-1.003)	0.524	1.005 (0.997-1.020)	0.833	1.002 (0.999-1.006)	0.207
SLM, 1 kg increase	0.928 (0.890-0.967)	<0.001	0.947 (0.885-1.013)	0.111	0.860 (0.774-0.957)	0.005
FFM, 1 kg increase	0.931 (0.895-0.968)	<0.001	0.949 (0.891-1.011)	0.103	0.867 (0.784-0.959)	0.005
SMM, 1 kg increase	0.876 (0.820-0.935)	<0.001	0.885 (0.791-0.991)	0.035	0.777 (0.655-0.922)	0.004
PBF, 1 percent increase	0.940 (0.908-0.975)	0.001	0.947 (0.901-0.996)	0.034	0.897 (0.841-0.956)	0.001
BCM, 1 kg increase	0.886 (0.834-0.940)	<0.001	0.895 (0.808-0.991)	0.034	0.794 (0.679-0.928)	0.004
VFA, 1 cm^2 increase	0.986 (0.974-0.998)	0.018	0.991 (0.977-1.006)	0.230	0.969 (0.947-0.992)	0.009

SMI, 1kg/m^2 increase	0.656 (0.509-0.845)	0.001	0.868 (0.576-1.309)	0.500	0.453 (0.257-0.799)	0.006
ICW, 1L increase	0.840 (0.771-0.916)	<0.001	0.852 (0.735-0.987)	0.033	0.718 (0.574-0.899)	0.004
ECW, 1L increase	0.849 (0.745-0.968)	0.014	0.980 (0.801-1.198)	0.841	0.690 (0.500-0.953)	0.024
TBW, 1L increase	0.912 (0.865-0.961)	0.001	0.940 (0.863-1.024)	0.154	0.829 (0.724-0.948)	0.006
ECW/TBW, 1% increase	1.943 (1.489-2.536)	<0.001	2.596 (1.654-4.075)	<0.001	1.529 (1.090-2.146)	0.014
PhA, 1 degree increase	0.533 (0.400-0.709)	<0.001	0.441 (0.284-0.687)	<0.001	0.671 (0.456-0.986)	0.042

Abbreviations: OR, odd ratio; 95 %CI, 95% confidence interval; HD, hemodialysis; WBC, white blood cell; RBC, red blood cell; AST, Aspartate aminotransferase; ALT, alanine aminotransferase; CRP, C-reactive protein; LDL-C, low-density lipoprotein cholesterol; HDL-C, high-density lipoprotein cholesterol; SLM, soft lean mass; FFM, fat free mass; SMM, skeletal muscle mas; PBF, percent body fat; BCM, body cell mass; VFA, visceral fat area; SMI, skeletal muscle mass index; ICW, intracellular water; ECW, extracellular water; TBW, total body water; ECW/TBW, extracellular water-to-total body water ratio; and PhA, phase angle

Supplementary Table S3: Correlations between independent continuous variables with p<0.05 in the univariate logistic regression and GNRI in overall subjects (*n* = 184)

Parameter	GNRI	Hb	Hct	Urea	Creatinine	TG	HDL-C	SLM	FMM	SMM	PBF	BCM	VFA	SMI	ICW	ECW	TBW	PhA	ECW/ TBW
GNRI	1																		
Hb	0.201	1																	
Hct	0.192	**0.920**	1																
Urea	0.186	0.006	0.014	1															
Creatinine	0.326	0.010	0.002	**0.616**	1														
TG	0.282	0.074	0.038	0.023	-0.173	1													
HDL-C	-0.190	-0.019	-0.013	0.022	-0.045	**-0.668**	1												
SLM	0.373	-0.011	0.010	-0.011	0.339	-0.110	-0.167	1											
FMM	0.367	-0.010	0.012	-0.008	0.342	-0.118	-0.161	0.999	1										
SMM	0.400	0.008	0.022	0.006	0.361	-0.087	-0.172	0.995	0.993	1									
PBF	0.298	0.040	0.008	0.037	-0.174	0.367	-0.305	-0.368	-0.363	-0.365	1								
BCM	0.400	0.008	0.022	0.007	0.362	-0.088	-0.171	0.995	0.992	0.999	-0.366	1							
VFA	0.245	-0.003	-0.018	-0.034	-0.235	0.306	-0.331	-0.198	-0.189	-0.221	**0.919**	-0.222	1						
SMI	0.381	-0.108	-0.084	-0.050	0.307	-0.107	-0.089	**0.934**	**0.929**	**0.930**	-0.372	**0.930**	-0.238	1					
ICW	0.402	0.010	0.024	0.007	0.361	-0.086	-0.173	0.995	0.992	0.999	-0.365	0.999	-0.221	**0.930**	1				
ECW	0.302	-0.080	-0.047	-0.049	0.269	-0.156	-0.160	0.967	0.971	0.943	-0.345	0.943	-0.120	**0.902**	0.943	1			
TBW	0.362	-0.016	0.006	-0.015	0.331	-0.121	-0.163	0.999	0.999	0.992	-0.368	0.992	-0.191	**0.934**	0.992	0.974	1		
PhA	0.386	0.188	0.140	0.188	0.281	0.217	0.043	0.152	0.135	0.227	-0.134	0.227	-0.374	0.174	0.228	-0.045	0.128	1	
ECW/TBW	-0.398	-0.231	-0.180	-0.210	-0.336	-0.208	0.046	-0.145	-0.128	-0.228	0.101	-0.228	0.361	-0.143	-0.229	0.062	-0.119	-0.907	1

Abbreviations: GNRI, geriatric nutritional risk index; Hb, hemoglobin; Hct, hematocrit; TG, triglyceride; HDL-C, high-density lipoprotein cholesterol; SLM, soft lean mass; FFM, fat-free mass; SMM, skeletal muscle mass; PBF, percent body fat; BCM, body cell mass; VFA, visceral fat area; SMI, skeletal muscle mass index; ICW, intracellular water; ECW, extracellular water; TBW, total body water; ECW/TBW, extracellular water-to-total body water ratio; and PhA, phase angle.
